# Differences in the attitudes to sport psychology consulting between individual and team sport athletes

**DOI:** 10.1186/s13102-021-00271-7

**Published:** 2021-04-29

**Authors:** Dáire Rooney, Robin C. Jackson, Neil Heron

**Affiliations:** 1 School of Medicine, Dentistry and Biomedical Sciences, Queen’s University Belfast, Belfast, Northern Ireland, UK; 2 School of Sport, Exercise & Health Sciences, Loughborough University, Leicestershire, LE11 3TU UK; 3 Centre for Public Health Research, Queen’s University, Belfast, Northern Ireland, UK; 4 UKCRC Centre of Excellence for Public Health Research (NI), Belfast, Northern Ireland, UK; 5 Department of General Practice, Keele University, Keele, England

**Keywords:** Sport psychology, Individual, Team, Attitudes, Stigma

## Abstract

**Background:**

The purpose of the present study was to investigate how an athlete’s participation in either an individual or team sport is related to their attitude toward sport psychology consulting and their willingness to consult a sport psychology practitioner.

**Method:**

The Sport Psychology Attitudes-Revised form (SPA-R) (Martin, et al., Sport Psychol 16:272-90, 2020) was completed by 120 athletes from individual and team sports. A 2 (Type of sport: individual and team) × 2 (Gender) multivariate analysis of variance (MANOVA) was conducted with attitudes towards sport psychology as dependent variables. To identify attitudes that accentuated the differences related to type of sport, follow-up univariate analyses were performed.

**Results:**

Results revealed that overall athletes involved in individual sports reported more positive attitudes towards sport psychology consulting than athletes involved in team sports. In particular, the athletes involved in individual sports were more likely to have greater confidence in sport psychology consulting. The findings also show that gender may mediate this association, indicated by a nearly significant two-way interaction effect for gender and type of sport (individual versus team) regarding confidence in sport psychology. The source of this marginal result was a larger effect of sport type for females than for males.

**Conclusions:**

The findings of this study imply that athletes involved in individual sports are more likely to have positive attitudes towards sport psychology compared to athletes competing in team-based sports, with females more likely to view sport psychology positively than compared to their male counterparts. The results may go some way to assist sport psychologists to understand and address athletes’ concerns and to improve receptivity to sport psychology services.

## Background

Advances in the science of sport performance increasingly demonstrate the importance of integrating mental attitude and physical skills [1, 2]. Such empirical evidence has led to the development of sport psychology as an integral aspect of coaching and health care for teams and athletes [3, 4]. Sport psychology can help an athlete to perform at a level closer to their absolute potential on any given day [5], and this has seen a marked increase in the number of sport psychology consultants working with athletes [6, 7]. Despite the apparent acceptance of the sport psychologist as a member of the ‘team behind the team’, receptiveness among athletes to sport psychology varies [8, 9]. Research into the factors that influence athletes’ attitudes towards sport psychology consulting (SPC) has largely focussed on individual athlete characteristics [10], type of sport [11–13] and perceived attributes and stigma [13].

Martin et al. surveyed collegiate athletes in order to determine the dimensions of athlete attitudes that accounted for differences in attitudes towards sport psychology [10]. The findings suggest that there are four main factors that determine attitudes towards sport psychology. The first is the “stigma tolerance”, which can be defined as the belief that an individual will be perceived negatively if they were to engage in psychology consulting [13]. The second factor is the athletes’ individual confidence in sport psychology consulting, based on their individual belief that this form of consulting will be beneficial in terms of improving mental skills and performance [14]. Thirdly, individual cultural preferences are thought to be a major factor. For example, Naoi et al. suggested that due to exposure to a much more ethnically and racially diverse society throughout their lives, American athletes showed less concern about working with consultants of different races and cultures than Japanese athletes [15]. Similarly, Ong and Harwood reported that Western athletes had less stigma toward sport psychology consulting, greater personal openness, and less preference for a consultant of the same race or culture than Eastern athletes, despite some Eastern countries being racially diverse, such as in Singapore [16]. The final factor is one’s personal openness, which represents the athlete’s degree of willingness to engage in sport psychology consulting and discuss relevant issues [14]. A study by Wrisberg et al. found that positively perceived sport psychology experiences amongst elite level student athletes led to more openness to future consultations [17].

Based on the above factors, Martin et al. developed a scale, known as the Sport Psychology Attitudes-Revised (SPA-R) to objectively assess an athlete’s expectations of, and receptiveness to sport psychology [14]. This validated questionnaire measures an athlete’s attitudes towards sport psychology based on four subscales that may explain differences in receptivity towards sport psychology. These subscales are: Confidence in Sport Psychology Consulting; Stigma Tolerance; Personal Openness; and Cultural Preference.

Subsequently, researchers have attempted to determine whether various groups differ in their mean scores on each subscale. Athlete gender has been demonstrated to play a role in determining attitudes and receptivity toward sport psychology, with studies consistently finding that, in comparison to females, males are less likely to seek psychological consulting [10, 18] and more likely to rely on themselves to deal with psychological issues [19, 20]. This finding is reflective of studies in the general population that find that females are more likely to seek help with mental health issues than males [21, 22]. As well as gender, certain personality traits have been found to influence one’s attitude towards sport psychology. For example, a study by Ong and Harwood found that openness and conscientiousness were associated with positive attitudes towards sport psychology [16]. Other studies have shown that individuals who demonstrated high levels of extraversion displayed more positive attitudes towards psychological support [23, 24].

Additional researchers have found that the characteristics of the sport, as well as personal characteristics such as gender and personality, determine attitudes towards sport psychology. For instance, researchers have found that athletes playing contact sports are more likely to have fewer positive attitudes towards sport psychology than those competing in non-contact sports, [11–13]. This finding might be explained by the nature of contact sports, that demands athletes to accept the pain and hurt that comes with the sport, perhaps suggesting a decreased willingness to seek help from a sport psychologist.

However, despite the growing research into the factors that influence an athlete’s attitude towards sport psychology, there are no studies that use an evidence-based and validated questionnaire to measure the influence of whether an athlete’s sport is individual or team-based. One of the main differences between team and individual sports is the influence that teammates, or a lack of teammates, can have on the athlete’s performance and perception of the sport.

Past research has shown that team-sport athletes who engaged in sport psychology were judged less favourably by their teammates regarding team selection when compared to other teammates who alternatively sought help from coaches for similar issues [25]. This might be explained by the historical stigma attached to sport psychology and mental health. Likewise, other studies have found that individual sport athletes demonstrated a greater willingness to partake in mental coaching when it was seen as beneficial to their individual level of performance [17]. Arguably, this is because of the increased personal responsibility that an athlete in an individual sport has in comparison to a team sport athlete, where responsibility is shared across the team. For this reason, it is important to establish whether differences exist in athletes’ attitudes to sport psychology in team and individual sports so that we can identify and break down the barriers that explain such a discrepancy. By identifying the factors that influence an athlete’s receptiveness to sport psychology consulting, it is possible to address the perceived barriers to sport psychology, thereby making this service more accessible and attractive to all groups.

The aim of the current study is therefore to use an objective measure to determine whether there is a difference in the attitudes of individual and team sport athletes towards sport psychology. Additionally, to examine whether variability in attitudes to sport psychology between athletes from individual and team sports is influenced by gender, we recruited an equal number of male and female competitors in each sport.

## Method

### Research design

The study design was a cross-sectional survey of athletes, with information gathered via the administration of a previously validated and published questionnaire (The Sport Psychology Attitudes – Revised) [14]. Ethical Committee approval was granted by the internal Loughborough University ethics committee and all methods were performed in accordance with the institution’s set guidelines and regulations. Following this, athletes from individual and team sports were invited to take part in the study. A priori power analysis using G*Power (v. 3.1), indicated a total sample size of 100 participants would yield acceptable power of 0.82 (MANOVA global effects, groups = 4, response variables = 4).

### Participants and demographic information

The study sample included 120 participants, equally split between individual sport and team sport athletes. Team sports were defined as those that involved competition between two teams each with two or more players. Individual sports were defined as those that involved the individual competing as a sole athlete in the sport. The individual sports were athletics, tennis and badminton, while the team sports were basketball, football and hockey. These sports were chosen on the basis of the availability of a sufficient sample of experienced male and female performers within the University where the study was undertaken. All participants were from the United Kingdom and played at college, county or national level in their respective sports. While subjects were recruited from university-based teams and organisations, some were graduates and so the ages of participants ranged from 18 to 34 years (*M* = 21.6, *SD* = 2.82). The mean age for team sports was 23 for males and 22 for females, while the mean age for individual sports was 20 for females and 21 for males. There were 30 males and 30 females in the individual and team sport groups. Twenty athletes (10 males and 10 females) were randomly chosen from each of the individual sports of tennis (singles), athletics and badminton and each of the team sports of football, basketball and hockey. A breakdown of the general demographic and sport information for the participants is shown in Table 1. Of the 120 athletes involved in the survey, 32 (29%) had previous experience of consulting a sport psychologist, with 23 (19%) of these from individual sports, whereas 9 (8%) were from team sports.

**Table 1:** Demographic Information of Participants; Type of Sport and Gender

SPORT	Male	Female	TOTAL
**Individual**			
*Tennis*	10	10	
*Badminton*	10	10	
*Athletics*	10	10	60
***Mean age***	21.05 (*SD* = 3.25)	19.95 (*SD* = 2.55)	20.50 (*SD* = 2.90)
***Previous Sport Psychology Experience***			
Yes	11	12	23
No	19	18	37
**Team**			
*Football*	10	10	
*Basketball*	10	10	
*Hockey*	10	10	60
***Mean age***	23.35 (*SD* = 3.50)	22.05 (*SD* = 2.25)	22.70 (*SD* = 2.65)
***Previous Sport Psychology Experience***			
Yes	7	2	9
No	23	28	51

### Data collection and instrument

Coaches of the identified individual and team sports were contacted via telephone to gain permission for the researcher to access the study participants. Participants were provided with a study information sheet, describing the purpose of the study so that informed consent could be obtained prior to the administration of the questionnaires. All athletes signed informed consent forms once an explanation of the study procedures was given. Study questionnaires were then randomly distributed at training sessions to athletes willing to participate in the study. No athletes opted not to participate after reading the study information sheet and therefore the response rate was 100%. Information regarding the athletes’ attitudes toward sport psychology consulting was gathered using the Sport Psychology Attitudes - Revised form. The 25-item questionnaire consists of four subscales: (a) stigma tolerance, (b) confidence in sport psychology consulting, (c) personal openness, and (d) cultural preference. As cultural preference was not relevant to the current research question, the five items in this subscale were removed from the questionnaire, leaving 20 items. Participants rated each item on a 7-point Likert scale ranging from 1 (strongly agree) to 7 (strongly disagree). To decrease the chance of response bias, items in one of the subscales, ‘stigma tolerance’, was framed negatively. Accordingly, a higher score in this subscale (indicating stronger disagreement) is indicative of an athlete who is less likely to stigmatise sport psychology and therefore views it more positively. Conversely, a higher score in the “confidence in sport psychology consulting” and “personal openness” subscales indicate that the individual has more negative attitudes toward sport psychology.

### Data analysis

Total scores for each subscale of the SPA-R questionnaire were calculated by adding together the responses for each question in that subscale. Reverse scoring was used for the “confidence in sport psychology” and “personal openness” subscales so that a higher score was associated with a more positive attitude towards sport psychology for all subscales. Participants’ mean scores for each subscale of the questionnaire were then calculated and entered into a two-way Multivariate Analysis of Variance (MANOVA) in order to detect differences according to gender (male vs. female) and sport (individual vs. team) as well as the interaction of these factors. Within this, type of sport and gender were entered as independent variables and the mean scores of each of the three subscales of the SPA-R questionnaire (confidence in sport psychology consulting, stigma tolerance, personal openness) served as dependent variables. An alpha level of .05 was used for all statistical tests.

## Results

### Descriptive statistics

Table 2 shows the descriptive statistics (mean and standard deviation) for each subscale of the SPA-R questionnaire, by gender and type of sport.

**Table 2:** Descriptive Statistics for Each SPA-R Scale by Gender and Type of Sport

SPA-R Subscale	Gender	Sport	Mean	Std. Deviation	*N*
Confidence in SPC	Male	Individual	5.18	.87	30
		Team	5.01	1.11	30
		Total	5.09	.99	60
	Female	Individual	5.47	.80	30
		Team	4.64	.87	30
		Total	5.05	.93	60
	Total	Individual	5.33	.84	60
		Team	4.82	1.01	60
		Total	5.08	.96	120
Stigma Tolerance	Male	Individual	5.42	1.28	30
		Team	5.09	1.34	30
		Total	5.25	1.32	60
	Female	Individual	5.36	1.14	30
		Team	5.02	1.23	30
		Total	5.19	1.19	60
	Total	Individual	5.39	1.20	60
		Team	5.06	1.27	60
		Total	5.22	1.24	120
Personal openness	Male	Individual	3.76	1.09	30
		Team	3.49	1.05	30
		Total	3.63	1.07	60
	Female	Individual	3.82	.87	30
		Team	3.43	.92	30
		Total	3.62	.91	60
	Total	Individual	3.79	.98	60
		Team	3.46	.98	60
		Total	3.63	.99	120

### Analysis

#### Preliminary analysis

Researchers have shown that previous experience of sport psychology consulting was associated with attitudes towards sport psychology [17]. In order to test this, a one-way multivariate analysis of variance (MANOVA) was conducted with prior experience of sport psychology as the independent variable (experience vs. no experience) and the mean scores of each of the three scales of the SPA-R questionnaire (confidence in sport psychology consulting, stigma tolerance, personal openness) used as the dependent variables. Results of the MANOVA indicated an overall small, statistically non-significant difference in attitudes towards sport psychology between athletes with and without prior consulting experience, Wilks’ Lambda = .94, *F* (3, 116) = 2.31, *p* = .08, η^2^ = .06. Univariate output for each subscale of the SPA-R questionnaire revealed that athletes who had prior experience of sport psychology services were slightly less likely to stigmatise sport psychology *(M* = 5.61, *SD* = 1.06) compared to those with no prior experience (*M* = 5.06, *SD* = 1.28), *F* (1, 118) = 5.08, *p* = .03, η^2^ = .04.

#### Main analysis

In order to consider the effect of gender and type of sport on athlete attitudes towards sport psychology, a 2 × 2 Multivariate Analysis of Variance (MANOVA) was conducted.

The mean scores in all three subscales of the SPA-R questionnaire were higher amongst athletes in individual sports compared to athletes in team sports (see Table 2).

**Fig. 1:**
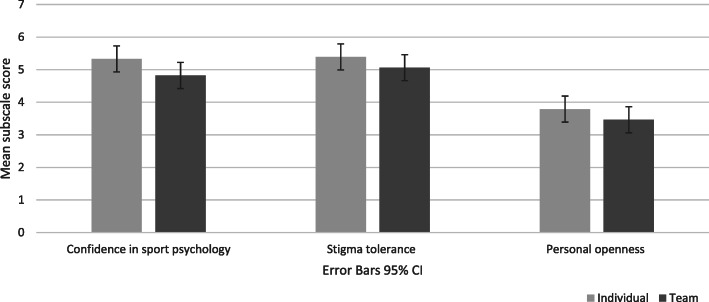
The effect of sport type on attitudes towards sport psychology in regard to: confidence in sport psychology consulting (CSP), stigma tolerance (STI), and personal openness (PO)

Results of the MANOVA indicated an overall difference in attitudes towards sport psychology between athletes of individual and team sports, Wilks’ Lambda = .89, *F* (3, 114) = 4.53, *p* = .01, η^2^ = .11. The univariate analyses indicated that there was a statistically significant main effect for type of sport on confidence in sport psychology consulting, *F* (1,116) = 8.77, *p* = .04, partial η^2^ = .07. This reflected that athletes from individual sports had greater confidence in the benefits of sport psychology consulting than athletes from team sports. The effect of type of sport on stigma tolerance was small and non-significant, *F* (1,116) = 2.14, *p* = .15, partial η^2^ = .02. The same was true for the personal openness subscale, *F* (1,116) = 3.35, *p* = .07, partial η^2^ = .03 (see Fig. 1).

**Fig. 2:**
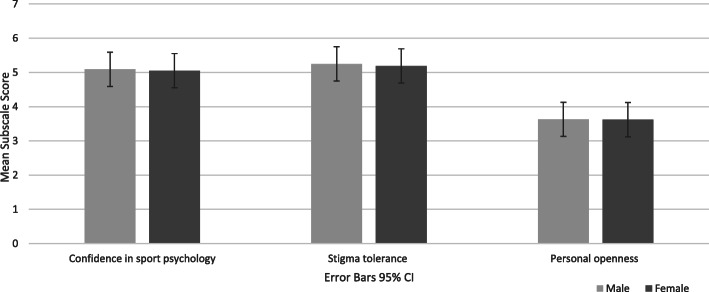
The effect of gender on attitudes towards sport psychology in regard to: confidence in sport psychology consulting (CSP), stigma tolerance (STI), and personal openness (PO)

Results of the MANOVA indicated no overall difference in attitudes towards sport psychology between males and females, Wilks’ Lambda = .99, *F* (3, 114) = .03, *p* = .99, η^2^ = .00. The univariate analyses confirmed non-significant effects for confidence in sport psychology [*F* (1,116) = .04, *p* = .84, partial η^2^ = .00], stigma tolerance [*F* (1,116) = .07, *p* = .79, partial η^2^ = .00], and personal openness [*F* (1,116) = .00, *p* = .99, partial η^2^ = .00] (see Fig. 2).

**Fig. 3:**
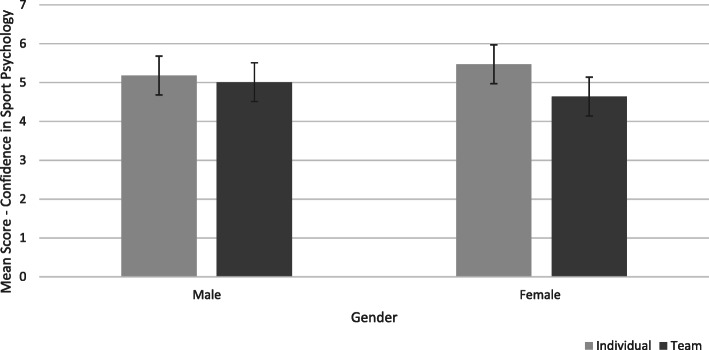
The interaction between gender and type of sport on the SPA-R subscale “Confidence in Sport Psychology Consulting”

Results of the MANOVA indicated a non-significant interaction between gender and type of sport, Wilks’ Lambda = .96, *F* (3, 114) = 1.42, *p* = .24, η^2^ = .04. The univariate analyses confirmed the interaction effect between gender and type of sport was non-significant for the three subscales of the SPA-R questionnaire. The interaction between gender and type of sport approached statistical significance for ‘confidence in sport psychology consulting’, *F* (1,116) = 3.89, *p* = .05, partial η^2^ = .03 (see Fig. 3). As can be seen in Fig. 3, the effect of sport type was slightly more pronounced for females than for males.

## Discussion

The current study aimed to assess and compare the attitudes towards sport psychology in male athletes and female athletes from individual and team sports. This study showed that athletes from individual sports had greater confidence in the benefits of sport psychology consulting than athletes from team sports. Gender was also an important factor in accessing sport psychology services - with individual male athletes and females in team sports less likely to engage in these support services. Additionally, athletes who had prior experience of sport psychology services were less likely to stigmatise sport psychology compared to those with no prior experience.

### Prior experience of sport psychology consulting

Individuals with prior experience of sport psychology had higher mean scores for each subscale of the questionnaire compared to those with no prior experience. This is no surprise given the vast amount of research that has shown that previous exposure to sport psychology is associated with positive attitudes towards this service [20, 26, 27]. Athletes without prior experience of sport psychology are likely to stigmatise sport psychology and thus view this service as equivalent to a more clinically based psychological consultation [5]. However, it is somewhat surprising that within the current study, stigma tolerance was the only dependent variable that was statistically significant. There are two possible reasons for this. Firstly, participants who have previously accessed sport psychology may have always been less likely to stigmatise this service, possibly due to certain personality traits or previous knowledge and experiences. Alternatively, athletes who initially stigmatised this service had their concerns allayed due to a positive experience with sport psychology.

### Type of sport

Results from the current study revealed differences in the attitudes towards sport psychology between athletes of individual and team sports. Individual sport athletes were more likely to have more positive attitudes towards sport psychology than athletes from team sports. Specifically, athletes engaged in individual sports reported higher mean scores in all three subscales of the SPA-R questionnaire compared to team sports athletes. In particular, there was a statistically significant effect for type of sport on confidence in sport psychology consulting, with individual sport athletes more likely to have greater confidence in the benefits of sport psychology. A possible explanation is that, in contrast to team sport athletes, individual sport athletes must rely fully on their own performance. They cannot count on teammates to compensate for any deficits, requiring them to have higher levels of preparation in order to optimise performance and increase likelihood of success [28]. The mental training component of this preparation requires them to develop a strong personal psychological focus. Thus, sport psychology might arguably be perceived as having particular benefit for the individual athlete, leading to greater belief in its value.

It is worth noting the potential for differences in how sport psychology consulting may be delivered between team and individual sports. Sport psychologists deliver their interventions in a number of ways, whether this be one-to-one or group settings. Team sport athletes may be less willing to pursue sport psychology due to a perception that this service is usually within a team setting and a fear of being embarrassed. It is therefore worth educating athletes on the wide array of sport psychology services.

### Gender

The current study showed a non-significant effect of gender but a small interaction between gender and type of sport in regard to confidence in sport psychology. The source of this was a larger effect of sport type in female athletes than male athletes. In short, this means that female individual sport athletes displayed a greater willingness to engage with sport psychologists in order to enhance performance compared to their male counterparts, whereas, in team sports, male athletes displayed greater willingness to engage with sport psychologists than female athletes. This suggests that gender differences in attitudes to sport psychology should be considered within the context of the individual or team environment in which they compete. This interplay was discussed by Coulter et al. [29] and Schinke & McGannon [30], who stressed the importance of considering an athlete’s personal traits and history in an attempt to disaggregate their attitudes about sport psychology. Indeed, the interaction highlights the danger of drawing inferences from very broad categorisations and illustrates the potential for larger-scale studies to provide a more fine-grained understanding of attitudes toward sport psychology.

### Methodological considerations and implications for future research

The study has a number of limitations that need to be borne in mind when considering the findings. Firstly, our main analysis did not directly account for the participants’ past experiences with sport psychology. Our preliminary analysis revealed that there was only one subscale with a statistically significant difference between athletes with prior experience of sport psychology services and athletes without prior experience and for this reason we opted not to include this in our main analysis. Despite this, it is still possible that a prior experience with sport psychology may have impacted the participants’ attitudes to sport psychology.

Secondly, the sample size was relatively small and so our study sample was not a random representation of all athletes, cultures and sports within the UK. Thirdly, participants either attended or had attended higher education, which may limit generalisability to less educated athletes. Set against this, the sample was balanced in regard to gender and sport type and was sufficiently powerful to detect moderate effects.

Lastly, the team sports chosen (football, hockey, basketball) are all considered to be “contact sports”, while the individual sports (athletics, badminton, tennis) are “non-contact” sports. Past studies have shown that there is an association between type of sport (contact vs non-contact) and athletes’ attitudes towards sport psychology, with athletes playing contact sports more likely to stigmatise sport psychology [11–13]. For this reason, effects attributed to sport type should be investigated in non-contact team sports (e.g., netball, volleyball) and/or individual contact sports (e.g., combat sports) in order to better understand the influence of these factors. There is therefore a clear need to confirm both the present findings across different sports and different educational backgrounds and to engage in larger-scale studies that allow for full consideration of a range of factors. Such research will also facilitate identification of possible mediating factors and more complex relationships.

## Conclusion

The current study considered athletes’ attitudes and openness to sport psychology consulting, with a view to identifying potential barriers to engagement with practitioners. In particular, it aimed to address whether there is a difference in the attitudes of individual and team sport athletes towards sport psychology, with the findings suggesting that individual athletes are more likely to have confidence in sport psychology services. The results also showed an interaction between gender and type of sport participation, with female individual sport athletes displaying a greater willingness to engage with sport psychologists in order to enhance performance, compared to their male counterparts. Whereas, in team sports, male athletes displayed greater willingness to engage with sport psychologists than female athletes although further research is required to confirm if these findings are consistent across ‘non-contact’ team sports compared to individual ‘contact/combat’ sports. These findings may prove important for sport psychologists wishing to maximise receptivity to their services within both team and individual sport settings. Indeed, sport psychologists working with individual male athletes and females in team sports, with no prior experience of sport psychology, need to be aware of the potential reluctance to engage with their services by these athletes and might spend more time educating them about the potential benefits to their sports performance.

## Data Availability

The datasets generated and/or analysed during the current study are available in the Loughborough University repository. This data is not readily available to the public.

## Abbreviations

SPC: Sport Psychology Consulting
CSP: Confidence in Sport Psychology
STI: Stigma Tolerance
PO: Personal Openness
SPA-R: Sport Psychology Attitudes - Revised form
